# Emerging Trends in the Use of Therapeutic Hypothermia as a Method for Neuroprotection in Brain Damage (Review)

**DOI:** 10.17691/stm2020.12.5.11

**Published:** 2020-10-28

**Authors:** E.Sh. Usmanov, M.A. Chubarova, Sh.Kh. Saidov

**Affiliations:** Researcher, Laboratory of Clinical Neurophysiology; Federal Clinical Research Centre for Intensive Care Medicine and Rehabilitology, 777 Lytkino Village, Solnechnogorsk District, Moscow Region, 141534, Russia; Junior Researcher, Laboratory of Clinical Neurophysiology; Federal Clinical Research Centre for Intensive Care Medicine and Rehabilitology, 777 Lytkino Village, Solnechnogorsk District, Moscow Region, 141534, Russia; Senior Researcher, Laboratory of Clinical Neurophysiology Federal Clinical Research Centre for Intensive Care Medicine and Rehabilitology, 777 Lytkino Village, Solnechnogorsk District, Moscow Region, 141534, Russia

**Keywords:** therapeutic hypothermia, neuroprotection, chaperones, cold shock hormones, cold shock proteins, controlled target temperature

## Abstract

The review analyzes current clinical studies on the use of therapeutic hypothermia as a neuroprotective method for treatment of brain damage. This method yields good outcomes in patients with acute brain injuries and chronic critical conditions. There has been shown the interest of researchers in studying the preventive potential of therapeutic hypothermia in secondary neuronal damage. There has been described participation of new molecules producing positive effect on tissues and cells of the central nervous system — proteins and hormones of cold stress — in the mechanisms of neuroprotection in the brain. The prospects of using targeted temperature management in treatment of brain damage are considered.

## Introduction

Medicine has been accumulating practical experience in the use of cooling agents for treatment and prevention of diseases during thousands of years. Hippocrates used snow and ice to stop bleeding in his patients [1]. In medieval times, hypothermia was used in the form of ice cubes to stop bleeding, in cases of cardiac arrest [2] and in comatose patients [3]. In the XIX century, Phelps used local head cooling in traumatic brain injuries [4, 5]. Whole-body exposure to cold was first used by neurosurgeon T. Fay in 1938 to treat head injuries. In the middle of the XX century, therapeutic hypothermia was forgotten due to increase in complications (bleeding, sepsis, heart rhythm disturbances) in the presence of general hypothermia when the patient’s body was exposed to deeper and longer cooling.

However, hypothermia has begun to develop again in the past three decades. This is a highly effective neuroprotective method used in various fields of modern medical practice.

The discovery of neuroprotection development mechanisms in hypothermia has attracted great interest [6]. The authors [6] showed the role of this method in management of many neurological diseases such as acute cerebrovascular accident (ACVA), traumatic brain injury, spinal cord injury, hepatic encephalopathy, and neonatal encephalopathy.

Hypothermia at a temperature of 33.5°C is the standard treatment for newborns with hypoxic/ischemic encephalopathy: it is applied in this category of patients for 72 h [7].

Nielsen et al. [8] were the first to introduce the concept of controlled target temperature, which comprises a wider temperature range (33–36°C) compared to therapeutic hypothermia and exerts a better effect on brain lesions. Moreover, the use of a target temperature has become increasingly widespread in cardiac surgery in patients with cardiac arrest to prevent anoxic brain damage [9]. Controlled target temperature in the range of 32–36°C for 24–48 h is one of the standard treatment algorithms in comatose patients after out-of-hospital cardiac arrest [10]. Preventive moderate and profound hypothermia also serves as standard management in cases of surgical interventions with possible cerebrovascular accidents such as cerebral aneurysms or in operations aimed to restore the aortic arch [11, 12]. In studies carried out at the Federal Clinical Research Centre for Intensive Care Medicine and Rehabilitology, great importance is attached to investigation of hypothermia in patients with chronic critical conditions. There have been observed clear positive effects exerted by this method on the level of consciousness in patients [13].

In clinical studies, Phadtare et al. [14] were the first to discover new molecules released through local and general body cooling — cold shock hormones (CSH) and cold shock proteins (CSP). They play an important role in the formation of progenitor cells for the nervous tissue of the brain, they are also necessary for repair and regeneration of damaged brain cells. The concept of “hypothermia in a syringe” has become a new trend in the development of therapeutic hypothermia [15]. It is based on neuroprotective cooling of individual organs and body systems while maintaining normal body temperature by intravenous administration of specific molecules inducing stress response to hypothermia and development of neuroprotection. This approach might have a wide range of applications: treatment of neurodegenerative diseases as well as acute and chronic critical conditions.

## Mechanisms of hypothermia-induced neuroprotection

**Figure 1:**
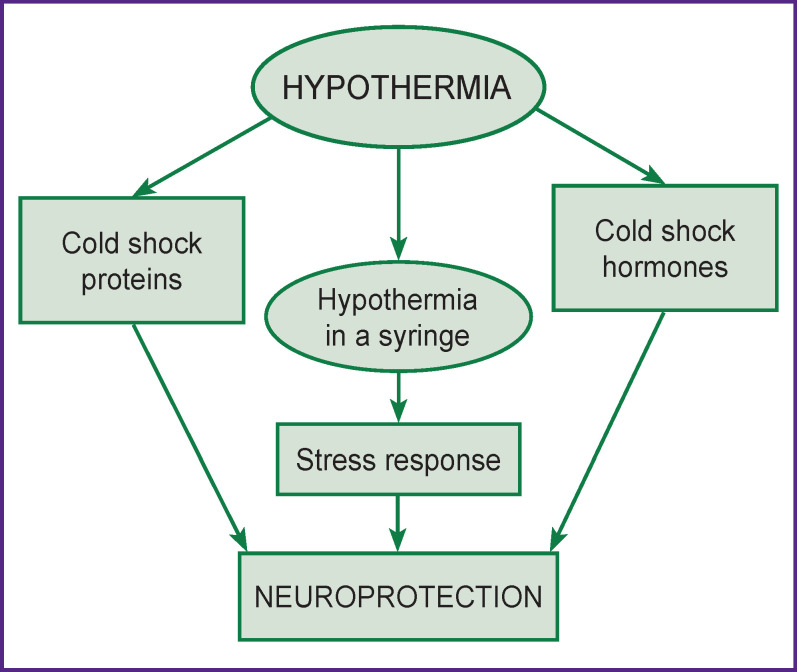
Neuroprotective mechanism in hypothermia

Therapeutic hypothermia is a promising method of neuroprotection against nerve cell damage. Its neuroprotective role has been best shown in experiments on dogs [16], rats [17], in patients with cardiac arrest [18], hypoxic/ischemic encephalopathies [19], traumatic brain injury [20], and some other diseases (Figure 1).

Despite the fact that hypothermic neuroprotection is still under research, its main mechanisms are likely to be a decrease in the level of nerve cell metabolism and free radical formation, reduced inflammatory changes, inhibition of excitotoxicity and apoptosis.

Various molecules may act as neuroprotectors. One of them is nestin, a neuroepithelial stem cell protein that belongs to cytoskeletal intermediate filaments. It was first described in the neural stem cells of the developing and developed brain. Nestin expression was found in stem cells of various tissues [21]. Notably, nestin-positive cells showing the ability to form neurospheres *ex vivo* and generate differentiated cells of the nervous and astrocytic lines are found in the brain [22].

β-tubulin III is another neuroprotective molecule, a microtubule element belonging to the family of tubulin proteins, one of the two main tubulins (α- and β-tubulins) required for heterodimerization and assembly of microtubules. This type of protein is found almost exclusively in nerve and testicular tissue. When expressed in neural tissue, β-tubulin III is involved in neurogenesis, axon guidance (the process of axon growth towards its target), and cell maintenance [23].

Cerebral ischemia causes neuronal damage (for example, after cardiac arrest or ACVA) and contributes to secondary damage after brain injury (especially in combination with hypoxia and/or hypotension) [24–26]. The amount of adenosine triphosphate (ATP) required by neurons to survive is proportional to the metabolic rate of the brain. During ischemia, the death of cells occurs due to the imbalance between the supply of cells with ATP (loss of oxidative phosphorylation in hypoxia) and their need for it (high oxygen consumption by brain tissues). In hypothermia, a decrease in temperature by each degree Celsius (from 37 to 27°C) is known to promote a decrease in oxygen consumption by brain tissues by 6–7% [27, 28]. Therefore, hypothermia is able to limit or prevent the development of ischemia during the episodes of impaired or completely absent blood flow in the brain due to decreased ATP consumption and the need to provide vitally important tissues with oxygen [29].

Oxidative stress contributes to tissue damage after traumatic brain injury due to increased production of toxic oxidation products (reactive oxygen species (ROS) and reactive nitrogen species (RNS)) and a decrease in the level of intracellular oxygen neutralization mechanisms. There are a large number of conditions for generation of ROS and RNS, including disturbances in the mitochondrial respiratory chain, activation of stimulating enzymes (xanthine oxidase, NADPH oxidase), and circulation of redox agents (free iron). The cumulative impact of these phenomena is exhibited as direct damage to proteins, lipids, and RNA/DNA [30]. Therapeutic hypothermia inhibits oxidative damage to the brain by reducing these processes [31–33] and enhancing antioxidant protection, which has been shown in clinical studies and in patients with various CNS injuries [34, 35].

Brain damage caused by excessive neuronal depolarization leads to intracellular Ca^2+^ overload and continuous production of glutamate (excitotoxicity) [36, 37]. Moreover, extracellular glutamate level also increases due to pathological changes in its astrocytic transporters [38]. The final effect of these events is rapid activation of extrasynaptic N-methyl-D-aspartate receptors, promoting intracellular apoptotic signaling cascade and subsequent neuron death [39]. Hypothermia potently inhibits neuronal death caused by direct incorporation of glutamate into the brain parenchyma [40]. In addition, brain cooling prevents posttraumatic surges of extracellular glutamate during ischemia [41, 42], brain concussions [31], subarachnoid hemorrhages [43, 44], and bacterial meningitis [45].

Release of intracellular DNA and dying cell debris into the extracellular space and secretion of additional damage-associated molecular patterns trigger production of pro-inflammatory cytokines (increased levels of TNF, INF-γ, and IL-6) [46, 47]. Moreover, neutrophils rapidly accumulate in the early stages after trauma and in the reperfusion phase after ischemia [48, 49]. The production of cytokines stimulates the pro-inflammatory (M1/M1-like) phenotype in macrophages and microglia [50, 51]. These events are a powerful mechanism of damage to CNS tissue. In the chronic phase, under ideal conditions, macrophages and microglia switch to the anti-inflammatory (M2/M2-like) phenotype, promoting recovery and lesion size reduction. However, recent studies have shown that the M2/M2-like phases reach their peak in the sub-acute and early chronic stages after brain injury, followed by a prolonged and negative phase of shifting towards the M1/M1-like phenotypes [52]. Hypothermia reduces neuronal inflammation by blocking the above triggers and shifting the monocytes towards the anti-inflammatory M2 phenotype [53, 54].

The blood-brain barrier (BBB) maintains the chemical composition of the brain interstitial fluid and is an essential structure required for the normal functioning of the central nervous system [55]. Increased BBB permeability due to mechanical damage after traumatic brain injury or in various pathological processes promotes penetration of pathogens and toxic micro/macromolecules into the underlying cerebral parenchyma [56, 57]. At the same time, migration of erythrocytes into the perivascular space with subsequent hemolysis leads to an increase in extracellular hemoglobin and free iron, aggravating the damage due to ROS [58]. Hypothermia reduces damage to the BBB in trauma [59, 60], ACVA [61, 62], bacterial meningitis [63], and intracerebral hemorrhage [64]. In hypothermia, protective mechanisms include inhibition of matrix metalloproteinases [65], preservation of proteins, tight junctions [66], reducing the level of intracellular ICAM-1 (cell adhesion molecule) on the vascular endothelial surface, they prevent diapedesis [59, 67].

The above injuries stimulate a variety of downstream signaling pathways that trigger different types of cell death mechanisms and lead to secondary brain damage. Each cell death mechanism has its own unique “molecular signature” including numerous effector molecules and signaling cascades. When using mild to moderate hypothermia, positive effects show as inhibition of the level of damaging enzymes or target molecules that trigger apoptosis [68, 69], necrosis [70], autophagy [64, 71], necroptosis [72], or pyroptosis [73, 74].

## Heat shock proteins

Various proteins released under the influence of stress on the body trigger cellular recovery mechanisms. Heat shock proteins (Hsp) are a class of reparative molecules. Therapeutic hypothermia is a powerful stimulus for Hsp production in brain cells. Exposure to low temperatures forms resistance of neurons to stress and stimulates progenitor cells, which in turn replenish and replace dead nerve cells.

Correct protein assembly process consisting of translation, transcription, and termination of the protein chain occurs in the presence of molecular chaperones, proteins involved in recognition and selective binding of foreign protein molecules, forming stable complexes [75]. They play an important role in protein assembly by preventing incorrect folding and aggregation of assembly components [76]. Hsp are molecular chaperones assisting the correct assembly of newly synthesized proteins as well as those susceptible to denaturation due to stress. In addition to their chaperone role, Hsp exhibit cytoprotective functions [77] and inhibit the apoptotic cascade [78].

Heat shock proteins are classified into different families based on molecular weight measured in kilodaltons (kDa): Hsp100, Hsp90, Hsp70, Hsp60, Hsp40, and smaller. Almost all families have representatives that play the role of constitutive proteins (produced irrespective of stress effects on the body cells) and inducible proteins (their synthesis is weak under normal conditions, but it increases sharply under stress effects on the cell). Hsp70 is the most studied chaperone promoting correct assembly and transfer of various protein molecules [79].

**Figure 2:**
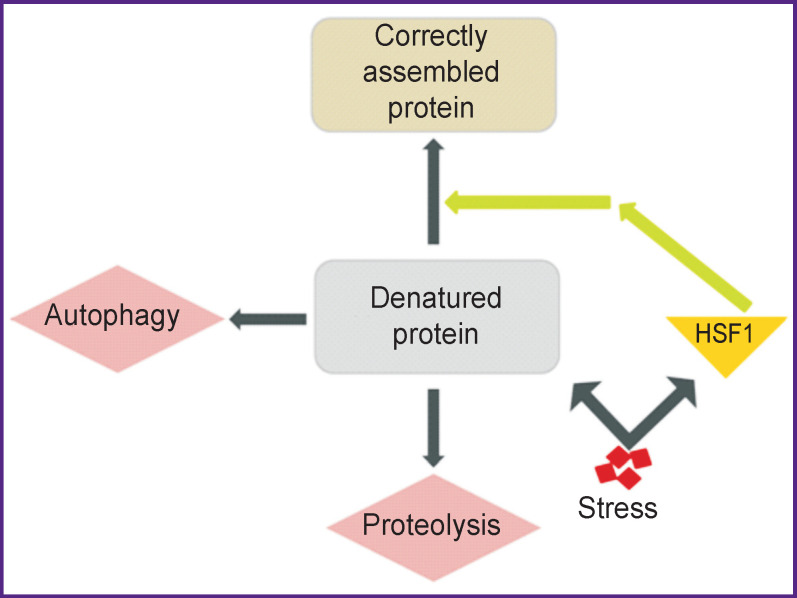
Induction of HSF1 and chaperones in response to stress

**Figure 3:**
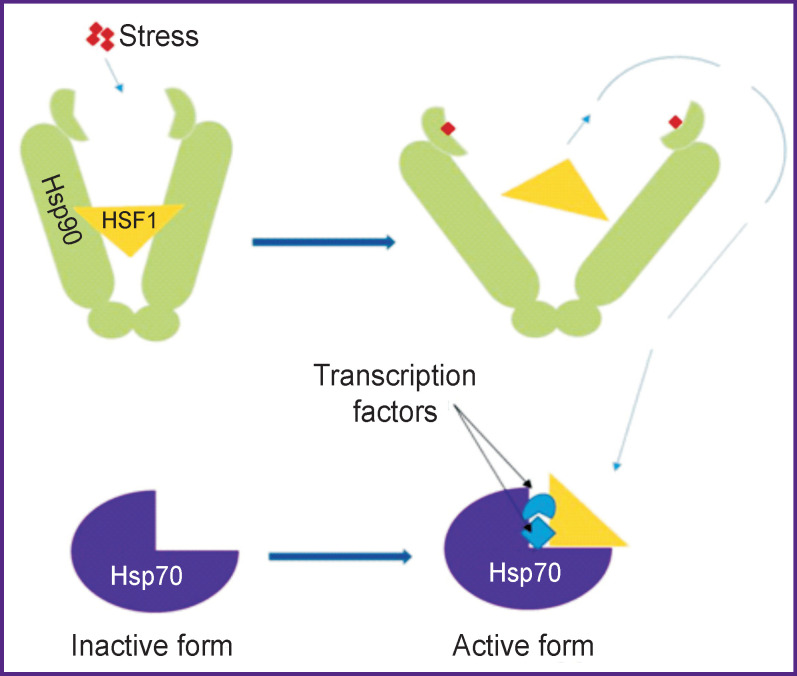
Release of HSF1 from the Hsp90 complex under the influence of stress and resulting activation of inducible Hsp70

The main transcription factor responsible for activation of heat shock proteins is heat shock factor 1 (HSF1) [80] (Figure 2). Under physiological conditions, it is associated with Hsp90, which inhibits its transcriptional activity, forming an inactive complex with it. Under stress conditions, released stress molecules bind to Hsp90 releasing HSF1, which in turn forms a bond with members of other heat shock protein families, inducing their expression [81]. Released from the complex, HSF1 migrates into the nucleus and binds Hsp gene promoters, which leads to increased regulation of these genes [80] (Figure 3).

Heat shock proteins are induced in the CNS by various pathological processes including ACVA, neurodegenerative diseases, epilepsy, and trauma [82]. Their expression is detected in various types of cells, including neurons, glia, and endothelial cells. They are also found as extracellular proteins formed by physiological secretory mechanisms and during cell necrosis. In the extracellular environment, these proteins increase resistance to stress by binding to stress-sensitive cells, including neurons [83].

When damaged, the brain becomes very vulnerable to even small temperature fluctuations [84]. Changes in the cellular environment of the brain during temperature stress involve formation of free radicals, changes in the mechanisms of nerve impulse transmission or a decrease in neuronal protein synthesis, and changes in gene expression.

Fluctuations in body temperature can lead to the death of brain cells and tissues (neurodegenerative changes) [85]. The necessary condition for repairing damaged brain cells is the presence of stem cells (progenitor cells). The potential of neural progenitor cells as a source of CNS tissue repair and regeneration was proved in work [86].

## Cold shock proteins and hormones

Along with its classic uses, hypothermia triggers mechanisms and events that function owing to production of cold shock hormones (CSH) and cold shock proteins (CSP).

Here, we consider the most studied ***cold shock hormones.*** Most fibroblast growth factors (FGFs) are paracrine hormones [87]. The proteoglycan-binding domain of heparin sulfate limits their activity up to complete shutdown [88]. In contrast, FGF21 is a member of the endocrine subtype, which includes FGF19, FGF21, and FGF23. Endocrine growth factors have lost their heparin-binding capacity during evolution, which allows them to circulate freely after being produced [89]. As a result, they use klotho transmembrane proteins as co-receptors (α-klotho and/or β-klotho) that play the role of molecular binders to facilitate and stabilize interaction between extracellular ligands and tissue receptors [90, 91]. The β-klotho protein is an obligatory co-receptor for FGF21 required for ligand binding and activation of the FGFR1c receptor *in vivo* [92, 93]. It was also shown that *in vitro* β-klotho protein increases affinity of FGF21 to bind to various isoforms of FGF receptors, but the magnitude of their activation depends on the type of receptor on target organs (FGFR1c > FGFR2c > FGFR3c) [92]. Moreover, β-klotho protein expression is limited to the following organs: liver, pancreas, adipose tissue, and some populations of hypothalamic and hindbrain neurons [94, 95].

Circulation of FGF21 increases in humans and rodents under cold stress. In humans, this phenomenon was proved in study [96], when patients wearing only hospital suits were left for 12 h in a ward with the surrounding temperature reduced to 19°C, after which the FGF21 level was measured in these patients and those in the wards with thermoneutral surrounding temperature (24°C).

Studies [97] show that an increase in FGF21 levels can improve brain function after acute pathologies and in chronic neurodegenerative conditions. The effects of FGF21 are believed to have direct and indirect mechanisms of action; it has also been revealed that FGF21 penetrates the BBB.

The direct effect of this factor was shown in work [97], where 5 nmol FGF21 was injected into samples for 6 days *in vitro*, which resulted in a decrease in damage to cells with FGF21 receptors due to glutamate. In addition, there was observed an increase in phosphorylation of neural AKT (AKT-1 kinase), ERK (extracellular signal-regulated kinases), and GSK-3β (glycogen synthase kinase), which lead to increased cell survival and neuroprotection. The authors of [98] argue that peripherally derived FGF21 promotes remyelination in the brain and spinal cord due to lysophosphatidylcholine. In study [99], it was found *in vitro* that FGF21 reduces damage to neurons induced by Aβ1–42 (a structural variant of β-amyloid) in patients with Alzheimer’s disease. It was also noted [100] that administration of FGF21 to normothermal subjects improved the BBB integrity, reduced cerebral edema and tissue damage, along with improvement in recovery from neurologic impairment. In [101], it was demonstrated that 14 days of therapy with 1.5 mg/kg of recombinant FGF21 (started 6 h after injury) reduced metabolic dysfunction, neuronal inflammation, reduced the area of cerebral infarction, white matter damage, and improved neurological results after focal ACVA. Finally, it was shown *in vivo* [102] that increased stress in the endoplasmic reticulum of brain neurons leads to phosphorylation of eukaryotic initiation factor 2 alpha (eIF2α), which in turn stimulates activation of transcription factor 4 (ATF4) and leads to an increase in neuronal expression of FGF21. The indirect mechanism of FGF21 action is stimulation of ketogenesis in the liver. Ketone bodies (acetone, acetoacetate, and β-hydroxybutyrate) are efficiently delivered to the brain where they serve as an alternative source of energy for oxidative metabolism [103].

Another mechanism of indirect action is the impact of FGF21 on the blood glucose level: this factor leads to its normalization [104]. However, unlike insulin, FGF21 normalizes blood glucose levels without inducing hypoglycemia [105]. Thus, stimulation or administration of FGF21 may be the best strategy for glycemic control in critically ill patients.

Irisin is a glycosylated protein fragment secreted by muscle tissue in response to training stress and muscle contraction (tremor) during cooling. The more contractions a muscle makes, the higher blood irisin level will be reached [106, 107].

Irisin is a neuroprotective hormone. It was found that intravenous introduction of 200 mg/kg of this substance 30 min after occlusion of the middle cerebral artery in ACVA model reduced the volume of cerebral infarction after 3 days [108]. It was revealed in another study [109] that irisin administration at a dose of 7.5 mg/kg directly into the ventricular system of the brain reduced the severity of neurological deficit, reduced infarction area, and edema of brain tissue. It was also observed that irisin influenced BDNF promoting its production and enhancing immunoreactivity. These phenomena lead to an increase in the neuroprotective properties of the brain and reduction of apoptosis. The impact of this hormone still requires further investigation, but it has already proved to be very promising as a factor for recovery and protection of neuroglia and astrocytes, which is worth investigating both in patients with acute and chronic critical conditions and in those with neurodegenerative diseases of the central nervous system.

Meteorin-like hormone was first identified as a factor of fat mobilization and PGC-1α4 expression (a hypertrophy regulator) in muscles. This hormone was found to have a stimulating effect on the anti-inflammatory function of macrophages due to the eosinophil-dependent expression of IL-4 [110]. CNS immune cells, including microglia and infiltrating macrophages, alter the extracellular microenvironment after brain damage [111]. Microglia and M1-type macrophages produce pro-inflammatory cytokines, while M2-type cells promote release of anti-inflammatory factors. Work [61] demonstrates that therapeutic hypothermia at 33°C for four hours increased the ratio of M2/M1 microglia and lymphocytes in the damaged cortex after concussion. Moreover, the authors of this study have shown that hypothermia increases expression of pro-inflammatory cytokines, including IL-10 and TGF-β.

Meteorin circulating in the blood activates anti-inflammatory pathways of macrophages, leading to their conversion into the M2 type.

***Cold shock proteins*** are produced during cold stress and stimulate cold adaptation in cells. After these proteins appear, they remain inside the cell and their level increases progressively with temperature decreasing below thermoneutral values. The therapeutic effect is achieved due to signaling pathway stimulation and leads to neuroprotection. We consider three clinically significant cold shock proteins: RNA-binding motif 3, cold-inducible RNA-binding protein, and Reticulon-3.

RNA-binding motif 3 (RBM3) was first described in study [112] demonstrating that cooling the body to 32°C for 24 h leads to an increase in RBM3 levels in mammals. Recent studies [113] have confirmed that hypothermia increases RBM3 levels in major neurons of the brain. An increase in RBM3 of messenger RNA (mRNA) was also observed in brain culture cells after incubation at 32°C for 72 h.

RBM3 is a potent neuroprotective agent. In patients with Huntington’s disease, there was observed a decrease in mRNA RBM3 in cells expressing toxic polyglutamine fragment HD-74Q. It has been shown [114] that the exogenous administration of RBM3 leads to inhibition of HD-74Q and reduces mortality of cells affected by this fragment.

**Figure 4:**
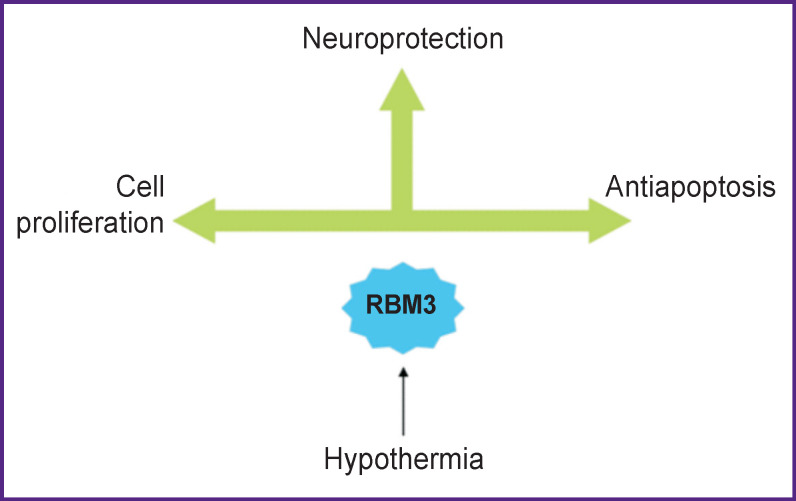
The main functions of the RBM3 protein

Increased RBM3 expression was also reported to reduce nitric oxide-induced cell death in neurons [115]. Delayed neuronal death is often observed in acute brain injuries [116, 117]. *In vitro* and *in vivo* experiments showed that production of RBM3 increased mRNA function, which in turn ensured survival of injured neurons [118, 119] (Figure 4).

Cold-inducible RNA-binding protein (CIRBP) as a representative of cold shock proteins was first discovered in the experiments of Nishiyama [120].

Modern *in vitro* and *in vivo* studies have found the level of CIRBP to increase in the main neurons of the brain under the influence of hypothermia. Studies by Li et al. [121] show that a decrease in cerebral cortex temperature to 32°C for 2 h increases CIRBP levels. Later Zhang et al. [122] demonstrated that exposure of cortical neurons to 32°C for 12 h also increased CIRBP. Similar results were obtained in the setting of complete body cooling, when an increase in CIRBP *in vivo* was recorded. Induced complete cooling of the body (31°C) in adult rats during 48 h promoted increased production of CIRBP in the hypothalamus [123]. Another study reports increased phosphorylation (activation) of the protective kinases pERK and pAKT due to CIRBP production after hypothermia [124]. Moreover, hypothermia increases release of protective proteins, including CIRBP, Bcl-2, and AKT, while decreasing apoptosis proteins such as Bax, Bad, Bak, caspase-3, caspase-9, and Apaf1 [125].

Neuroprotective properties of CIRBP have been revealed in cases of post-traumatic brain injury [123]. The level of this protein increased in the hypothalamus of rats after brain injury and remained elevated for 48 h after cooling to 31°C during 48 h. The same study has reported on decreased levels of apoptosis in damaged cells of the cerebral cortex, hippocampus, and hypothalamus.

Study [126] notes that hypoxia decreases proliferation of cells lacking cold-inducible RNA-binding protein. CIRBP blocks the death of these neurons. Hypothermia protects them by preventing apoptosis and reducing the function of targets for apoptotic proteins, including HIF1α.

The latest representative of cold shock proteins is Reticulon-3 (RTN3A1) first described by Moreira et al. in 1999 [127]. The authors of [128] showed that RTN3A1 is a cold shock protein as it is produced in neurons while cooling. This protein is a modulator of pathogenesis in Alzheimer’s disease. The precursor of β-amyloid BACE1 stimulates processing of the amyloid precursor protein (APP) into β-amyloid that forms blue plaques in the brain [129]. Inhibition of BACE1 is one of the possible treatment options for Alzheimer’s disease and RTN3A1 inhibits BACE1 via two mechanisms. The first is inhibition of axonal transport of BACE1 protein to synapses, which impairs its interaction with APP reducing formation of blue plaques; the second is a decrease in the number of neurotoxic fragments (Aβ1–40 and Aβ1–42) in the cerebral cortex [130].

## Conclusion

Therapeutic hypothermia is a powerful neuroprotective method affecting the brain through a variety of mechanisms. Promoting production of heat shock proteins, this method builds neuronal resistance to stress and in turn stimulates progenitor cells regenerating and replacing dead nerve cells. Therapeutic hypothermia generates cell differentiation of the nervous and astrocytic lines by stimulating production of nestin. Application of this method offers the possibility to limit or prevent development of ischemia in the absence of blood flow to the brain due to decreased ATP consumption and the need to provide vitally important tissues with oxygen. Produced during hypothermia, cold shock hormones and proteins have anti-inflammatory, neuroprotective, and stimulating effects on brain cells, which makes them one of the most important mechanisms triggered by therapeutic hypothermia.

The studies of recent decades offer us the opportunity to make sure of the immensity of proven effects obtained through therapeutic hypothermia, but at the same time, there is a huge field for discovering new possibilities of this method, manifested at the molecular level. It is still necessary to study, develop, and apply therapeutic hypothermia for treatment of patients in acute and chronic critical conditions.
